# Asia-Pacific malaria is singular, pervasive, diverse and invisible

**DOI:** 10.1016/j.ijpara.2016.06.006

**Published:** 2017-06

**Authors:** J. Kevin Baird

**Affiliations:** Eijkman Oxford Clinical Research Unit, Jakarta, Indonesia; Centre for Tropical Medicine and Global Health, Nuffield Department of Medicine, University of Oxford, Oxford, United Kingdom

**Keywords:** Malaria, Diversity, Asia-Pacific, Control, Elimination, Strategy, Tools

## Abstract

Malaria in the Asia-Pacific region has been targeted for elimination by the year 2030. This article asks the question, “by what means?” in the context of proven technical strategies and tools against key challenges imposed by the distinct character of the Asia-Pacific malaria problem. The misperception of malaria in the Asia-Pacific region as a less serious but otherwise essentially similar problem to African malaria lulls us into rote application of the same tools and strategies. Those now mitigating the harm done by malaria in Africa will not suffice to eliminate malaria in the Asia-Pacific region – these tasks and the problems are fundamentally distinct. This article describes the singular characteristics of Asia-Pacific malaria and the bearing of those upon the technical strategy of malaria elimination. Most of the tools needed for that endeavour do not yet exist and spirited calls for elimination within the next 14 years may discourage the patience and investments needed to conceive, optimise and validate them.

## Highlights

• Asia-Pacific malaria has unique character and diversity.
• Over 2 billion residents of the Asia-Pacific region live at risk of malaria infection.
• The tools to eliminate malaria in the Asia-Pacific region do not yet exist.
• Combatting malaria in the Asia-Pacific region is likely to extend well beyond 2030.

## Graphical abstract

**Figure:**
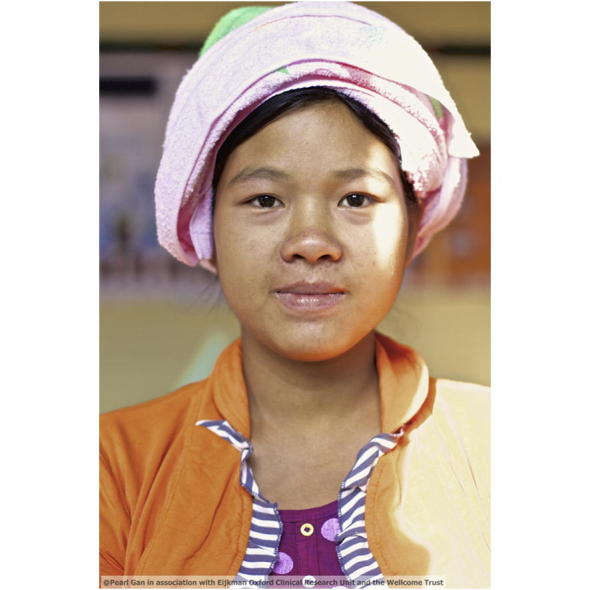

## 1 Eliminating malaria

The diverse community engaged in mitigating the harm done by malaria – and to ultimately eliminate it – focuses on the extraordinary burdens of morbidity and mortality caused by *Plasmodium falciparum* on the African continent. The technical strategies for doing so now implemented there came with compelling bodies of scientific evidence demonstrating positive impacts. Indeed, recent estimates of morbidity and mortality across Africa point to profound progress having been achieved in the past decade (Bhatt et al., 2015). These gains occurred after the extraordinary scaling up of specific evidence-based interventions – rapid diagnostic tests (RDTs), artemisinin combined therapies (ACTs), long-lasting insecticide-treated bed nets (LLINs), and indoor residual insecticide spraying (IRS) (Smithson et al., 2015). In addition to the less tangible but vitally important assets of social awareness, community engagement, political will, and historic generosity, those commodities compose the physical tools against malaria. Moreover, the research community has endeavoured for over 40 years to field a vaccine aimed at protecting highly vulnerable African infants and small children from poor outcomes of infection. The most advanced vaccine, RTS, S, reduced the risk of febrile clinical attacks of relatively high parasitemia, but not infection per se or death (Greenwood and Doumbo, 2016).

In the African context RDTs, ACTs, LLINs and IRS surely mitigate harm, and a vaccine that prevents illness and death would save very many lives. However, few of these tools rationally align with strategies to wholly eliminate malaria. In a tactical and strategic sense, mitigating the harm of high transmission is quite distinct from eliminating its causative agents where unstable or low transmission prevails. This distinction may be lost upon non-technical advocates for eliminating malaria, but it is crucially important to grasp.

In 2007 Bill and Melinda Gates implored the malaria community to adopt a strategy of eliminating malaria rather than learning to live with it in perpetuity by mitigation of harm alone (Tanner and de Savigny, 2008). The World Health Organization (WHO) embraced and formalised the concept in 2008, and later wisely specified epidemiological contexts where elimination goals may be realistically achieved. Those appropriately excluded much of holo-endemic sub-Saharan Africa and high transmission areas in southern and southeastern Asia. Heavily malarious areas first must reduce transmission to low levels before considering elimination as a goal of defined milestones. In contrast, the many nations in the Asia-Pacific region having unstable or hypo- to meso-endemic malaria seemed suited to an elimination agenda. By 2009 many had formally adopted elimination goals and joined the Asia-Pacific Malaria Elimination Network (Gosling et al., 2012). In 2013 representative heads of state formed the Asia-Pacific Leaders Malaria Alliance, declaring the elimination of malaria from the Asia Pacific by 2030 as their collective goal (http://aplma.org/blog/24/East-Asia-Summit-leaders-endorse-APLMA-Malaria-Elimination-Roadmap/).

## 2 Quo Pacto?

This Latin phrase asks, “by what means?” The meaning of that question in the context of this article does not refer to the social and political assets needed to rally human energies and fiscal resources to the cause of eliminating malaria in the Asia-Pacific region. Instead, it is a technical question referring to the tangible tools, tactics and strategies aimed at specific biological problems to be engaged in that enterprise. If the important task of rallying support for elimination is successful, how will those mobilised resources be translated into significant gains against malaria in the Asia-Pacific region? What tools will be brought to bear and how? This article addresses these questions in the context of crucially important differences between the malaria problem of Africa and that of the Asia-Pacific region. In brief, the tools that have successfully mitigated harm in Africa (RDTs, ACTS, LLINs, IRS) are poorly suited to the task of eliminating malaria in the Asia-Pacific region. Malaria here imposes obstacles to elimination not shared with African malaria and few tools yet exist to cope with those.

The elimination agenda must recognise and acknowledge important biological distinctions between the African and Asian-Pacific malaria problems. The means of eliminating malaria from the Asia-Pacific region will necessarily be fundamentally distinct from those of mitigating harm done by the relatively heavy burdens of malaria in much of Africa. What are those distinctions and by what means will humanity drive the plasmodia to extinction in the Asia-Pacific region? These questions will also be relevant across the very long and wide fringes of the heavily malarious heart of Africa – areas where there is relatively low and more diverse ecologies of transmission.

## 3 Singular, pervasive, diverse and invisible

The perception of malaria in the Asia-Pacific region as an essentially similar but less serious problem than African malaria oversimplifies it to an extraordinary degree. The two problems are wholly distinct in biological, clinical, epidemiological and social dimensions. Few commonalities link them beyond the role of anopheline mosquitoes in conveying the plasmodia amongst humans. A lay view of that limited commonality imposes the dangerous risk of rote adoption of African strategies and tools in driving an Asian-Pacific malaria problem to elimination. Understanding specific features of Asia-Pacific malaria that distinguish it from the dominant high burden African malaria is key to formulating a rational elimination strategy.

The singular nature of Asia-Pacific malaria is its enormous diversity and range relative to African malaria in terms of species of parasites, their mosquito vectors, epidemiology, ecology of transmission, parasite resistance to drugs, human genetics and awareness of malaria as a serious problem. The following summarise these distinctions.

### 3.1 Geographic distribution and burdens

Endemic malaria transmission occurs in almost all nations of the Asia-Pacific region, excepting only Japan, Australia, New Zealand and Singapore (Gething et al., 2012), and Sri Lanka recently arrested endemic transmission (Fernando et al., 2015). Summing up the populations living at risk of infection in those areas – with exclusion of the many interspersed geographic areas known to be malaria-free – yields 2 billion versus 2.8 billion for all of humanity (Gething et al., 2011). Transmission in the majority of those areas is unstable or hypo-endemic – present but causing relatively few cases per person-year compared with the intense holo-endemic transmission occurring in much of sub-Saharan Africa. The burdens of morbidity and mortality across the vast endemic expanse of the Asia-Pacific region are exceedingly difficult to credibly estimate.

A study of malaria-attributable mortality in India illustrates the uncertainties (Dhingra et al., 2010). Systematic verbal autopsy of 100,000 randomly selected deaths in a single year suggested 205,000 people lost their lives to malaria, 86% of them away from treatment centres, in areas where diagnosis and reporting was highly improbable. In that same year, the WHO estimated only 15,000 deaths due to malaria in India (Hay et al., 2010). The report stirred controversy (Butler, 2010) and many in public health rejected the findings as too extraordinary to be credible and cited analytical pitfalls of verbal autopsy. The important point on mortality due to malaria in the Asia-Pacific region is that uncertainty – we don’t know the absolute burdens of morbidity and mortality linked to malaria in the Asia-Pacific region. Either tens of millions of infections occur with the loss of tens of thousands of lives, as estimated by the WHO (World Health Organization, 2015, World Health Organization, 2015), or several hundred million cases occur with the loss of a few hundred thousand lives as implied by the Indian mortality study.

The relatively low levels of transmission in the Asia-Pacific region should not lull us into believing or hoping that absolute morbidity and mortality burdens are also relatively low – the enormous numbers of people composing denominators of risk for illness or death may conceal very substantial numbers of people composing the numerators, the ill and dead.

### 3.2 Low intensity transmission

The task of bringing low transmission to zero is far more challenging than reducing high levels of transmission. A number of key factors help explain this. High prevalence of microscopically- or RDT-patent infection of blood is the rule in high transmission settings. Meta-analyses consistently reveal an inverse correlation between the proportion of sub-patent to patent parasitemias and prevalence of infection (Cheng et al., 2015, Moreira et al., 2015, Wu et al., 2015). As prevalence diminishes, the proportion of sub-patent infections rises. The sensitivity of diagnostic methods declines with the level of transmission. The exhaustive surveys of 7355 residents of rural areas of Thailand, Cambodia and Vietnam by Imwong and colleagues (2015a) recorded prevalence at 4% by RDT, 5% by expert microscopy and 20% by a highly sensitive PCR technique. This problem of a highly prevalent asymptomatic and sub-patent reservoir imposes one of the great challenges for elimination – case detection and treatment cannot achieve impacts if only one in five case is detected. The impact of mass screen and treatment interventions wanes as the undiagnosed and untreated sub-patent reservoir represents larger proportions of those infected.

Without far more sensitive point-of-care diagnostic methods than microscopy or current RDT technologies, targeted mass drug administration campaigns may be necessary to attack this key reservoir of infection and transmission (WHO, 2016). Those campaigns, however, require more intensive surveillance activities than are typically carried out, i.e., conducting the campaigns where and when needed is by no means a trivial task (WHO, 2010). Perhaps most important in the Asia-Pacific setting, the inclusion of hypnozoitocide for mass treatment is not currently practical and treatment strategies excluding that therapy have little or no impact on the prevalence of *Plasmodium vivax* (Maude et al., 2014). It may be implausible in a social sense to approach communities with the promise of eliminating a particular kind of malaria but not all of them. In a public health strategic sense, attacking malaria one species at a time is not sensible because it multiplies the energies and resources required.

### 3.3 Dominance of *P. vivax*

Asia-Pacific malaria is dominated by *P. vivax*, and the region accounts for >80% of the global burden (Mendis et al., 2001). Although *P. vivax* occurs all across Africa, Duffy factor negativity apparently suppresses transmission and prevalence. Relative to the African *P. falciparum* problem, *P. vivax* on that continent occurs as a public health problem only along the northern edges of the Sahel, across the Horn, and the island of Madagascar (Howes et al., 2015). The intense focus of humanity on the extraordinary African malaria problem, and the relatively minor role of *P. vivax* in it, in part explains the chronic neglect of this infection in malariology over the past 60 years (Mendis et al., 2001, Baird, 2007, Price et al., 2007). Another very important factor in that neglect was the erroneous assignment of an intrinsically benign character to the malaria caused by *P. vivax*. Evidence accrued over the past decade affirms an often pernicious course with severe and fatal outcomes associated with severe anaemia, severe thrombocytopenia, respiratory distress, renal or hepatic dysfunction, seizures/coma and shock (Anstey et al., 2012, Baird, 2013). Thus, even in the Asia-Pacific region, *P. vivax* has been deeply neglected as a scientific, clinical and public health problem.

Specific features of the biology of *P. vivax* make it a more daunting problem than *P. falciparum*. It is less likely to be diagnosed by an RDT (WHO, 2012). It is widely resistant to first-line therapy with chloroquine in the Asia-Pacific region (Price et al., 2014). Its hypnozoites are widely tolerant of standard primaquine therapy against them (John et al., 2012), and strains in the region exhibit some of the most aggressive relapse behaviours (Battle et al., 2014). Moreover, extreme sensitivity to primaquine by variants of relatively severe inherited glucose-6-phosphate dehydrogenase (G6PD) deficiency disorder in humans dominates all across the Asia Pacific region (Howes et al., 2012, Howes et al., 2013). Another distinct problem with *P. vivax* is the inability to offer primaquine therapy against relapse to 93 million at-risk and highly vulnerable pregnant women and their infants, together with several hundred million G6PD-deficient patients living at risk (Dellicour et al., 2010, Howes et al., 2012). These patients will require other treatments or chemo-preventive drugs against relapse.

Strategies aimed at eliminating *P. falciparum* have virtually no impact upon *P. vivax* because they exclude attack of a hypnozoite reservoir absent in *P. falciparum*. In one recent study at the Thai-Myanmar border, an estimated 96% of clinical attacks by *P. vivax* originated from the latent liver stages (Adekunle et al., 2015), as did >80% of clinical vivax malaria in a Papua New Guinea study (Robinson et al., 2015). Inability to safely attack or manage the enormous threat imposed by the hypnozoite reservoir of *P. vivax* in the Asia-Pacific region bodes poorly for prospects of elimination (Tanner et al., 2015). Doing so will require mobilising G6PD point-of-care diagnostics to the periphery of care wherever this infection occurs (Baird, 2015) and to conceive, optimise and validate strategies for preventing relapses in those unable to receive hypnozoitocidal therapies.

### 3.4 Artemisinin-resistant *P. falciparum*

The problem of artemisinin-resistant *P. falciparum* emerged on the Thai-Cambodian border a decade ago and now occurs all across the Greater Mekong Sub-Region (http://www.who.int/malaria/publications/atoz/status-rep-artemisinin-act-resistance-sept2015.pdf?ua=1). Vigorous efforts to contain or eliminate this problem have not been successful. ACT treatment failures now commonly occur in Thailand, Cambodia, Laos and Vietnam (Imwong et al., 2015b). In the public health arena, artemisinin resistance is acknowledged as a dire threat to the African continent, now almost wholly reliant upon ACTs and without a replacement therapy at the ready (White, 2010). The urgency of containment or elimination in the region mobilised very substantial resources aimed at doing so (Kolaczinski et al., 2014). The continuing spread and deepening of artemisinin resistance, while threatening potentially catastrophic loss of life at some point in the future in Africa, threatens lives and elimination ambitions in the Asia-Pacific region today. Alternative emergency therapies such as atovaquone-proguanil are being applied to achieve cure, but the susceptibility of that drug to rapid and complete onset of resistance by a single point mutation in its target molecule renders it an extremely thin line of defense against untreatable falciparum malaria. Mitigating the harm done by *P. falciparum*, much less its elimination, requires new therapies of proven and lasting efficacy against artemisinin-resistant strains.

### 3.5 Zoonotic malarias

Infection of humans by the plasmodial parasite of southeastern Asian macaques, *Plasmodium knowlesi*, is known to occur throughout most of the region (Moyes et al., 2014). Most confirmed cases have come from case reports of travellers, but in Malaysian Borneo where the problem was first described over a decade ago (Singh et al., 2004), robust epidemiological studies revealed it as the dominant cause of malaria cases (Singh and Daneshvar, 2013). Similarly thorough studies have yet to be undertaken elsewhere in the region, largely for want of the substantial funding needed to do so. The expertise needed is widely available in the region, but the will and resources needed to examine the problem are not.

Critical questions remain unanswered regarding the malaria zoonoses of southeastern Asia: (i) How widespread is the problem? (ii) Are asymptomatic carriers present? (iii) Are other species involved? (iv) Is human-to-human transmission of these species occurring? In clinical studies done during the 1950s and 1960s, experimental infections of humans with not only *P. knowlesi* but also *Plasmodium cynomolgi*, *Plasmodium inui*, and *Plasmodium fieldi* (all natural parasites of macaques, amongst others in southeastern Asian monkeys and apes) successfully infected humans who then infected mosquitoes that became infective to both humans and monkeys (Coatney et al., 1971). These plasmodia also infect a variety of anopheline species and not solely those occurring naturally in the forest habitats of macaques (Coatney et al., 1971). Biologically, human-to-human transmission in the wild is thus perfectly plausible and perhaps even likely given that confirmed vectors of human plasmodia in southeastern Asia also become naturally infected by the monkey malaria species (Maeno et al., 2015). Human-to-human transmission of these parasites may well be occurring.

A single case report of a human naturally infected by *P. cynomolgi* (Ta et al., 2014) illustrates the vital importance of a great deal more work on this problem. This species is very closely related to *P. vivax*, including latency with dormant hypnozoites. Under the microscope, it is morphologically indistinguishable from *P. vivax*, and in that reported case reacted positively to standard nested PCR primers for *P. vivax*. Absent the sequencing of those PCR amplicons, the diagnosis would have been confirmed as *P. vivax*, and absent more thorough investigations of scale, the proportion of human *P. vivax* cases being *P. cynomolgi* remains an important unknown. The scale of an animal reservoir of malaria in humans is a conspicuously important and almost wholly neglected question in the elimination agenda for the Asia-Pacific region.

### 3.6 Diversity of vectors and transmission ecology

Whereas a few species of anopheline mosquitoes of largely shared ecologies and behaviours (dominated by the anthrophilic and endophilic *Anopheles gambiae*, *Anopheles funestrus,* and *Anopheles arabiensis* complexes) occur in highly endemic Africa, several dozen confirmed vector species (many of them zoophilic and exophilic) flourish across the Asia Pacific region in an enormous variety of habitats and ecological settings (Sinka et al., 2011, Sinka et al., 2012). Starting with modest observational studies in the 1980s (Bradley et al., 1986), the next two decades saw the collection of hard evidence of diminished all cause mortality in Africa by use of insecticide-bearing bed nets (Choi et al., 1995, Lengeler, 2004). That evidence underpinned the massive distributions of many hundreds of millions of LLINs that then followed (from about 2005 onwards). Very few randomised controlled trials of LLINs have been done in Asia-Pacific region, and one of the most thorough reported modest or no protective effects (Smithius et al., 2013a). Those investigators attributed that outcome to observed and recorded early evening and outdoor feeding preferences by the anophelines present (Smithius et al., 2013b). Reliance upon LLINs to eliminate malaria in the Asia-Pacific region rests upon hope of efficacy rather than realised evidence.

### 3.7 Mobile and migrant populations

The relatively high density of human populations throughout much of the Asia-Pacific region drives people to seek economic opportunity away from their homes. That is, they are mobile and migrant, and the region has a tradition of this lifestyle reaching back centuries (http://www.migrationpolicy.org/article/migration-asia-pacific-region), including domestic migration. In India up to 100 million people may be mobile and migrant within borders, most of them urban poor seeking seasonal work in agriculture. Such movement, from a densely populated area of limited economic opportunity to relatively unsettled frontiers with endemic malaria transmission, may be typical of most seasonal migrations.

Taking Indonesia as an example, millions of Javanese seek economic opportunity amongst the still sparsely populated other islands across this 5300 km archipelago. Most retain links to Java and regularly return, sometimes bringing acute, sub-patent or latent malaria with them (Elyazar et al., 2011, Sutanto et al., 2013, Nelwan et al., 2015). A study of the movements of 91 million cellular phones on Java revealed 17 million travelling to other islands in Indonesia in a single year (Elyazar I, Eijkman-Oxford Clinical Research Unit, Indonesia, personal communication). Excepting sporadic outbreaks and a handful of stubborn low-intensity foci of endemic transmission, Java is virtually free of malaria (Elyazar et al., 2011). However, the island remains highly receptive to malaria with at least several dangerous anopheline vectors occurring in substantial numbers and across wide areas (*Anopheles sundaicus, Anopheles maculatus, Anopheles aconitus* and *Anopheles balabacensis* to name a few) (Elyazar et al., 2013). There is little doubt that most of the chronic outbreaks regularly occurring on Java derive from infected migrants. What is true for Indonesia is also regionally true – malaria elimination will require strategies for coping with the threat of malaria imported by travellers and migrants into highly receptive areas, within and between borders.

Those strategies have yet to be conceived, much less optimised and validated. The tracking and treating of these migrants before they transmit malaria seems highly implausible given the vast numbers and geography. One solution may be a disciplined and sustained implementation of anopheline species sanitation methods as a means of protecting malaria-free areas at high risk of importation. That forgotten art of malaria control achieved huge impacts in the Netherlands East Indies and many other locations before the age of IRS and LLINs (Takken et al., 1990, Keiser et al., 2005). The lethal malaria epidemic at the Segera Anakan estuary on the southern coast of Java in 1984 illustrates this danger: imported malaria and environmental conditions suited to *An. sundaicus* drove transmission (Atmosoedjono et al., 1992). The Asia-Pacific region may need to again apply anopheline species sanitation in order to prevent the reseeding of malaria-free areas by hundreds of millions of mobile and migrant people.

### 3.8 Vaccination strategy

Almost all work on malaria vaccines over the past decades has focused on preventing death in young Africans exposed to *P. falciparum*. Preventing infection per se fell away relatively early in that endeavour as evidence mounted, indicating inability to do so with available molecular subunit vaccine technologies. The sparing of life, by rendering infections less likely to progress to death, by effective vaccination is a sensible and worthy objective in the African context. In the Asia-Pacific region an intervention that exacerbates the asymptomatic reservoir problem may not be suited to elimination strategy. However, others argue that perhaps the RTS,S vaccine could be useful in the Asia Pacific region (Gosling and von Seidlein, 2016). A vaccine that prevents infection per se, i.e., sterilising protection, is needed. If available, such a product would likely find application in the problem of mobile and migrant workers, i.e., vaccination of those at high risk of returning with malaria, or of those resident in areas of high risk of reintroduced malaria. At present, the only vaccine technology holding out such promise is live attenuated sporozoite vaccines (Hoffman et al., 2015). These ought to be optimised and validated in the context of eliminating the Asia-Pacific malaria problem, including examination of the possibility of cross-species protection by a *P. falciparum* sporozoite vaccine (Sedegah et al., 2007).

### 3.9 Invisible malaria

The asymptomatic, sub-patent and latent reservoirs of malaria give the term ‘invisible malaria’ a physical reality. Those malarias dominate the epidemiological landscape of Asia-Pacific malaria. But there is an important social dimension to the term as well. In both lay and expert communities, the malaria problem of Asia-Pacific region pales in comparison to that of Africa. The extraordinary levels of transmission and exhaustive evidence documenting mortality amongst infants and small children in Africa rivet international humanitarian attention on that problem. In the Asia-Pacific region, malaria occurs amongst the most invisible people within the many societies affected and the problem often exceeds the public health capacities needed to take notice.

**Fig. 1:**
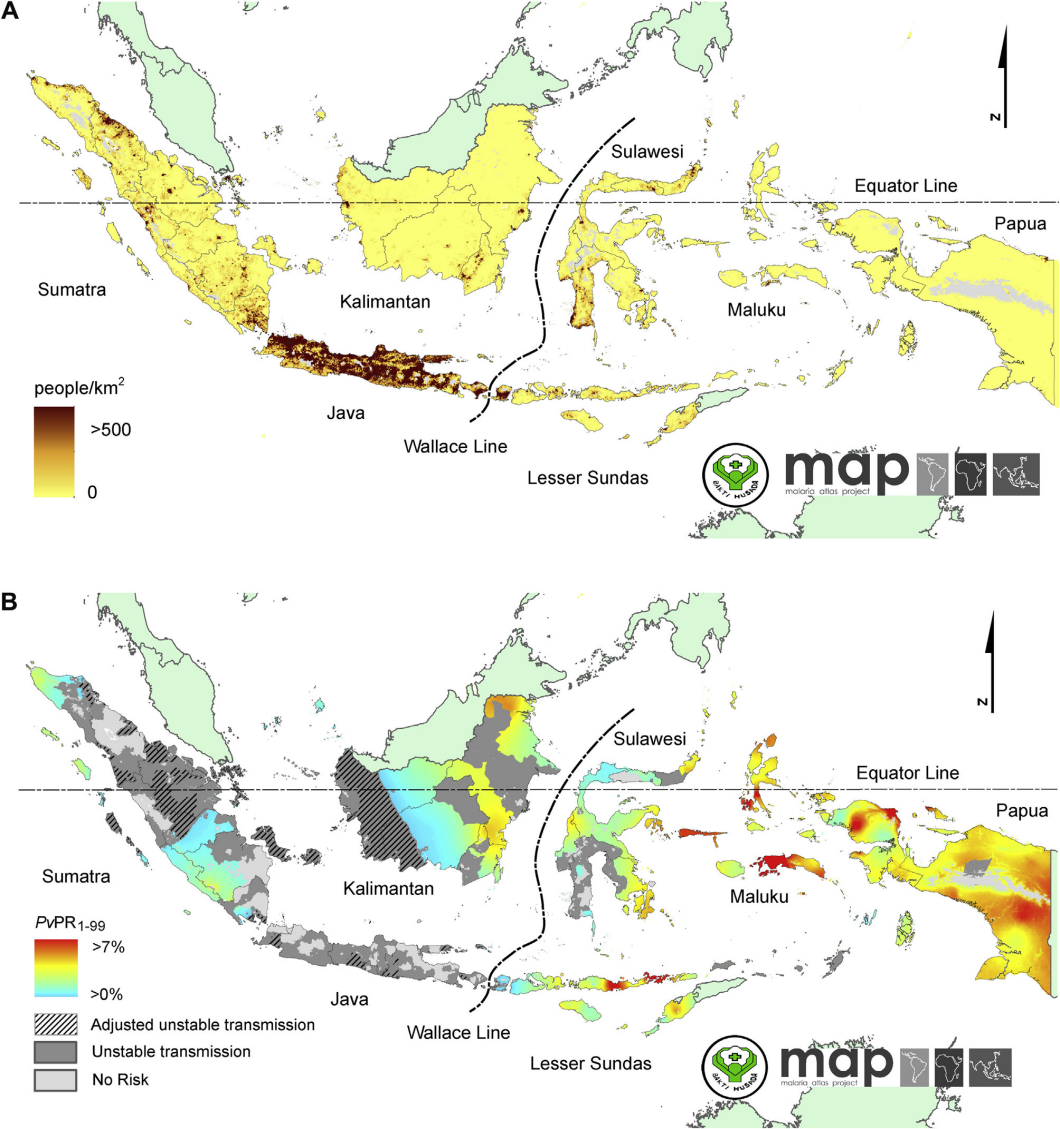
Maps illustrate inverse correlation of (A) population density in Indonesia (courtesy of Malaria Atlas Project, University of Oxford, UK and the Ministry of Health, Republic of Indonesia) with (B) risk of malaria (reproduced with permission from Elyazar, I.R.F., Getting, P.W., Patil, A.P., Rogayah, H., Sariwati, E., Palupi, N.W., Tarmizi, S.N., Kusriastuti, R., Baird, J.K., Hay, S.I., 2012. *Plasmodium vivax* malaria endemicity in Indonesia in 2010. PLoS One 7(5) e37325. doi: http://dx.doi.org/10.1371/journal.pone.0037325). The sparsely populated outer islands of the Indonesian archipelago are dominated by stable prevalent *Plasmodium vivax* (and *Plasmodium falciparum* (not shown)). The islands of Java and Bali are home to 150 million of Indonesia’s 250 million citizens, and have no stable prevalent malaria transmission.

The Asia-Pacific region was home to 4.3 billion people in 2013 (http://www.unescapsdd.org/files/documents/SPPS-Factsheet-Population-Trends-v3.pdf), 57% of them living in rural settings (http://www.unescap.org/stat/data/syb2011/I-People/Urbanization.asp). Although urban malaria transmission occurs in India, the responsible vector, *Anopheles stephensi*, is largely absent from the rest of region. No other anopheline species in the region tolerate urban environments. Asia-Pacific malaria is thus overwhelmingly a disease of those living in relatively isolated and impoverished rural areas. The demographic example of the Indonesian archipelago of 13,500 islands highlights this important fact (Fig. 1), where 150 million of its 250 million citizens live on just two islands, Java and Bali. Whereas malaria transmission on Java and Bali is sporadic, isolated to very few low-grade foci, and nearly eliminated, all of the other islands have stable endemic transmission in most rural areas. Providing robust health services and surveillance all across the sparsely populated archipelago poses steep challenges to the government. As in most developing nations, the rural poor often live beyond the reach of routine clinical care and the systems that monitor, count and report what ails them. The mortality study from India (Dhingra et al., 2010) confirmed this by finding 86% of deaths occurred away from treatment centres. Morbidity and mortality, and its causes, go largely without investigation and reporting by severely stretched and limited clinical and public health services. Epidemiologically sound surveillance of morbidity and mortality and its causes may be essentially absent across large swathes of the rural Asia-Pacific region.

The absence of robust estimates of malaria morbidity and mortality across most of the Asia-Pacific region lulls us dangerously to accept it as evidence of relatively light absolute burdens amongst the two billion people living at risk. The widely disregarded study of malaria mortality in India (Dhingra et al., 2010) warns us to gather materialised evidence. That report of an estimated 205,000 deaths due to malaria in India alone evoked criticisms of methodologies and broad dismissal of the findings, thereby preserving the status quo of the absence of evidence. An appropriate refutation of seemingly scientifically valid study findings of such gravity would be the materialised evidence of scientifically more robust mortality estimates of an order of magnitude lower. Failing to do so, simple disbelief and dismissal registers as irrational confidence or blind ignorance.

## 4 Implications for elimination

Eliminating malaria from the Asia-Pacific region requires specific technical strategies and tools for coping with all of the unique features of the problem summarised here. Unfortunately, most of these strategies or tools have not yet been conceived and none have been optimised and validated. Table 1 summarises these requirements. Given the slow pace of even vigorously supported clinical research agendas with bright prospects endowed by existing technologies, the vision of a malaria-free Asia-Pacific region by 2030 seems highly implausible in a technical sense. Some of the key required technologies do not yet exist, e.g., a true point-of-care diagnostic device offering PCR-like sensitivity of detection of infection. Firm commitment to elimination in the Asia Pacific region will require a great deal more patience and investments in developing the technologies and tools needed, their optimisation and validation, and finally their systematic implementation in robustly managed and executed programmes of elimination. This requires acknowledging the inadequacy of African solutions that mitigate that problem and recognising the unique requirements for malaria elimination in the Asia-Pacific region. Absent such, the singular, pervasive, diverse and invisible problem of malaria in the Asia-Pacific region will be a clinical and public health battleground for generations to come.

**Table 1:** Suite of tools needed for the elimination of malaria in the Asia-Pacific region.

Elimination tool	Utility	Status
Therapy for artemisinin-resistant *Plasmodium falciparum*	Eliminating *P. falciparum*	Phase II and III studies underway
Single-dose therapy against hypnozoites of *Plasmodium vivax*	Attacking the hypnozoite reservoir	Phase III studies (tafenoquine)
Point-of-care G6PD diagnostic device	Safe access to hypnozoitocidal therapy	At least one kit commercially available and promising validation data (CareStart G6PD™ (AccessBio®, USA))
Chemo-preventive strategies against relapse without access to hypnozoitocidal therapy	Managing relapse risk in pregnant women, young infants, G6PD deficient patients, and CYP2D6-disabled patients	Not conceived
Point-of-care diagnostic for all species of *Plasmodium* with PCR-like sensitivity	Attacking the asymptomatic/sub-patent reservoir	Several technologies in early development
Surveillance of malaria as a zoonosis	Understanding the threat posed by an animal reservoir for human malaria	Accomplished only in Malaysian Borneo
Species sanitation of anophelines	Rendering malaria-receptive areas less vulnerable to imported malaria	Technology abandoned over 70 years ago, almost entirely not practiced
Surveillance and healthcare delivery capacities reaching the most rural and isolated populations	Access to monitoring and care by the most at-risk populations	Limited by economics, technical capacities and geography
Sterilising protective vaccination	Dealing with mobile and migrant population risk of reintroduced malaria	Live attenuated sporozoite vaccines in phase II development

The danger in spirited advocacy for elimination of Asia-Pacific malaria by 2030 is the arrest of the hard work of developing the very tools needed to achieve it. Young scientists in the region may steer clear of what is perceived as a vanishing health problem. Funding agencies may find investment in long-term development of tools unattractive or irrational – a new class of hypnozoitocides, for example, may take a decade or more of relatively very expensive work, only to emerge when presumably no longer needed. If the year 2030 arrives and the plasmodia have yet to be eliminated from the region, we may find deferred investments in people and technical progress will have ensured the inability to be rid of this problem.

Malaria is a formidable foe, especially in the Asia-Pacific region. A vision of eliminating the plasmodia that excludes the technical means of doing so should be acknowledged as dangerous hubris. These parasites will not surrender to human will alone, but to human ingenuity aimed at highly specific technologically defined points of deliberate and sustained biological attack on them. Conceiving those strategies and tools, optimising and validating them, and finally aggressively implementing them should be the focus of the earnest will to drive the plasmodia to extinction in the Asia-Pacific region.
